# Acupuncture and other traditional Chinese medicine therapies in the treatment of children’s tic syndrome: A network meta-analysis

**DOI:** 10.3389/fnins.2023.1156308

**Published:** 2023-04-17

**Authors:** Tong Pu, Yu Liu, Junxia Wang, Jieying Zhang, Jinhao Zhang, Zhiling Ran, Qiaoni Sheng, Zhiqiang Yi, Jiansong Ye, Yanling Li, Xijun Wang, Hao Chi, Wei Luo

**Affiliations:** ^1^College of Acupuncture and Tuina and Rehabilitation, Hunan University of Chinese Medicine, Changsha, China; ^2^Department of Oncology, Chongqing General Hospital, Chongqing, China; ^3^Department of Pediatrics, The Affiliated TCM Hospital, Southwest Medical University, Luzhou, China; ^4^First Teaching Hospital of Tianjin University of Traditional Chinese Medicine, Tianjin, China; ^5^National Clinical Research Center for Chinese Medicine Acupuncture and Moxibustion, Tianjin, China; ^6^School of Stomatology, Southwest Medical University, Luzhou, China; ^7^BaZhong Hospital of Traditional Chinese Medicine, Bazhong, China; ^8^Bazhou District People’s Hospitals, Bazhong, China; ^9^Department of Rehabilitation Medicine, People’s Hospital of Ganluo, Liangshan Yi Autonomous Prefecture, China; ^10^Rosemead College, Rosemead, CA, United States; ^11^School of Basic Medical Sciences, Xianning Medical College, Hubei University of Science and Technology, Xianning, China; ^12^Clinical Medical College, Southwest Medical University, Luzhou, China; ^13^Affiliated Traditional Chinese Medicine Hospital of Southwest Medical University, Luzhou, China

**Keywords:** stroke, children’s tic syndrome, neural mechanism, acupuncture, acupuncture and moxibustion, network meta-analysis

## Abstract

**Background:**

Tic disorders (TD) are a kind of neuropsychiatric disease that frequently occur among preschool and school-age children, mainly characterized by motor tics or sometimes accompanied by vocal tics, and its pathogenesis is still unclear. The clinical manifestations are mainly characterized by chronic multiple movements, rapid muscle twitching, involuntary occurrence, and language disorder. Acupuncture, tuina, traditional Chinese medicine, and other methods are commonly used in clinical treatments, which have unique therapeutic advantages but have not been recognized and accepted by the international community. This study conducted a quality evaluation and meta-analysis of the currently published randomized controlled trials (RCTs) of acupuncture for TD in children in order to provide reliable evidence-based medical evidence for acupuncture for TD.

**Methods:**

All the randomized controlled trials (RCTs) using the intervention methods acupuncture + traditional Chinese medical herbs, acupuncture + tuina, and acupuncture, and the control group using Western medicine were included in the analysis. The main outcomes were obtained by using the Yale Global Tic Severity Scale (YGTSS), the Traditional Chinese medicine (TCM) syndrome score scale, and clinical treatment efficiency. Secondary outcomes included adverse events. The risk of bias in the included studies was assessed according to the tool recommended by Cochrane 5.3. The risk of bias assessment chart, risk of bias summary chart, and evidence chart in this study will be produced using R and Stata software.

**Results:**

There were 39 studies that met the inclusion criteria, including 3,038 patients. In terms of YGTSS, the TCM syndrome score scale changes and shows a clinically effective rate, and we found that acupuncture combined with Chinese medicine is the best treatment.

**Conclusion:**

Acupuncture + traditional Chinese medical herbs may be the best therapy to improve TD in children. At the same time, compared with Western medicine commonly used in clinical practice, acupuncture and acupuncture combined with tuina therapy have better effects on improving TD in children.

## 1. Introduction

Children’s tic disorder is a common movement disorder in childhood, and its incidence has gradually increased in recent years (Knight et al., 2012; Cubo et al., 2017). Its prevalence rate among preschool children has reached as high as 6.1% (Yang et al., 2016), with a male-to-female ratio of about 4:1 (Robertson et al., 2009). The prevalence of this disease in children and adolescents is 0.1–0.6%, and the male-to-female ratio is (3–4):1. After treatment, approximately two-thirds of the children can achieve control or symptom relief, and approximately one-third of the children’s symptoms still fluctuate and can continue to adulthood. Clinical symptoms are mainly manifested as multiple, involuntary muscle production in one or more parts, and repeated rapid motor twitch and vocal twitch (Freeman et al., 2000). Sometimes, attention deficit hyperactivity disorder (ADHD), obsessive–compulsive disorder, and other mental complications can be accompanied clinically, thus increasing the clinical treatment difficulty of children with TD (Scharf et al., 2012; Zinner et al., 2012).

Currently, in the Western medicine treatment system, the main treatment methods for children with tic disorders are oral Western drugs such as haloperidol, thioperide, and risperidone (Weisman et al., 2013). Although these drugs have certain curative effects, children and their parents are more inclined to use non-drug therapy due to adverse reactions such as drowsiness and nausea and the instability of the curative effect (McHugh et al., 2013; Mogwitz et al., 2013).

At present, a large number of studies have shown that perinatal adverse factors may lead to the onset of TD in children (Pasamanick and Kawi, 1956; Bos-Veneman et al., 2010; Hoekstra et al., 2013). Bad habits are also an important factor leading to the onset of TD. According to previous studies, cola, coffee, black tea, preservatives, and sweeteners can directly or indirectly affect the dopamine system in the brain, thereby causing or aggravating tic symptoms in children (Müller-Vahl et al., 2008). At the same time, with the development of social science and technology, the long-term use of mobile phones, televisions, and computers leads to the long-term exposure of children to radiofrequency radiation and magnetic field radiation, which may change the function of the central nervous system by changing the structure of nerve cell membranes (Maier et al., 2004). Other studies believe that the incidence and recurrence of TD in children may be partly associated with the infection of pathogenic microorganisms. Studies have found (Krause et al., 2012) that the decline of immune function and the abnormal production of autoantibodies after infection in some children are caused by the imbalance of the autoimmune system. Repeated viral or bacterial infection is an important cause of immune dysfunction and autoimmune pathological damage. Generally, simple TD only damages the subcortical tissue structure such as the basal ganglia, but the hyperactivity disorder caused by TD may also cause damage to the frontal lobe, resulting in patients being more prone to problems such as attention deficit or aggression.

Traditional Chinese medicine theory related to the disease is often relegated to emotion, diet, innate endowment, and external sensation. Although there is no significant organic disease and damage, the motor twitch and vocal twitch caused by this disease seriously affect the growth and development of children, life and learning, and mental state. According to the traditional Chinese medicine theory, the treatment of this disease is mainly related to “jiang huo” (reduce the fire in the body), “xi feng” (extinguish the wind inside), “hua tan” (reduce phlegm in the body), and other treatment methods such as acupuncture and other traditional Chinese medicine means of comprehensive treatment, the effect is very significant.

## 2. Methods

### 2.1. Protocol and registration

The protocol of this systematic review and meta-analysis is registered in PROSPERO, under the registration number CRD42022376370. It is available from the following website.^1^

### 2.2. Inclusion criteria

The studies included in this study should be randomized controlled trials (RCTs). In the treatment group, acupuncture combined with tuina, acupuncture combined with traditional Chinese medical herbs, and simple acupuncture (needle materials, acupoints, and techniques do not limit the treatment means) were included. The control group was treated with Western drugs that were more commonly used in clinical practice, including but not limited to haloperidol, thioperide, and risperidone.

### 2.3. Exclusion criteria

In this analysis, we excluded the following criteria: (1) non-randomized controlled trials; (2) trials without baseline data assessment; (3) animal experimental studies, reviews, meeting minutes, case reports, or expert experience summaries, and other non-randomized controlled trial studies; (4) the number of people in the experimental group and the control group was significantly different; (5) usage of the blank control test; (6) in the same experiment, the two groups had the same type of intervention, such as millimeter needle treatment in both groups; (7) only abstract available and the lack of full text or important information report is incomplete and the contact author did not respond; (8) the original text is not standard, and there are obvious mistakes.

### 2.4. Outcomes

#### 2.4.1. Primary outcomes

1. Motor tic, vocal tic, and tic total scores on the Yale Global Tic Severity Scale (YGTSS);
2. The changes of nodding and shrugging, the voice in the larynx, upset, and main symptom scores in the Traditional Chinese medicine (TCM) syndrome score scale.

#### 2.4.2. Secondary outcomes

1. Clinical effective rate;
2. Adverse reactions.

### 2.5. Literature search

The literature search time of this study was from the establishment date of each database to 15 November 2022, and the following databases were searched: China Biology Medicine, China National Knowledge Infrastructure, Wan Fang Data, the Chinese Science and Technology Periodical Database, Medline, Excerpt Medical Database (EMBASE), Web of Science, and the Cochrane Library, which has no national or language restrictions. The search strategy includes a combination of free-text terms, synonyms, and subject headings related to Tourette syndrome in children and its associated subjects (Table 1).

**Table 1:** Search strategy.

No.	Literature search term
1	randomized controlled trial [pt]
2	controlled clinical trial [pt]
3	randomized [tiab]
4	placebo [tiab]
5	clinical trials as topic [mesh:noexp]
6	randomly [tiab]
7	trial [ti]
8	#1 OR #2 OR #3 OR #4 OR #5 OR #6 OR #7
9	animals [mh] NOT humans [mh]
10	#8 NOT #9
11	tic disorder [mesh]
12	tourette syndrome [tiab]
13	#11 OR #12
14	acupuncture [mesh]
15	acupuncture therapy [mesh]
16	massage [tiab]
17	tuina [tiab]
18	herb [tiab]
19	#14 OR #15 OR #16 OR #17 OR #18
20	#8 AND #13 AND #19
21	remove duplicates from #20

### 2.6. Study selection and data extraction

#### 2.6.1. Selection of studies

**Figure 1:**
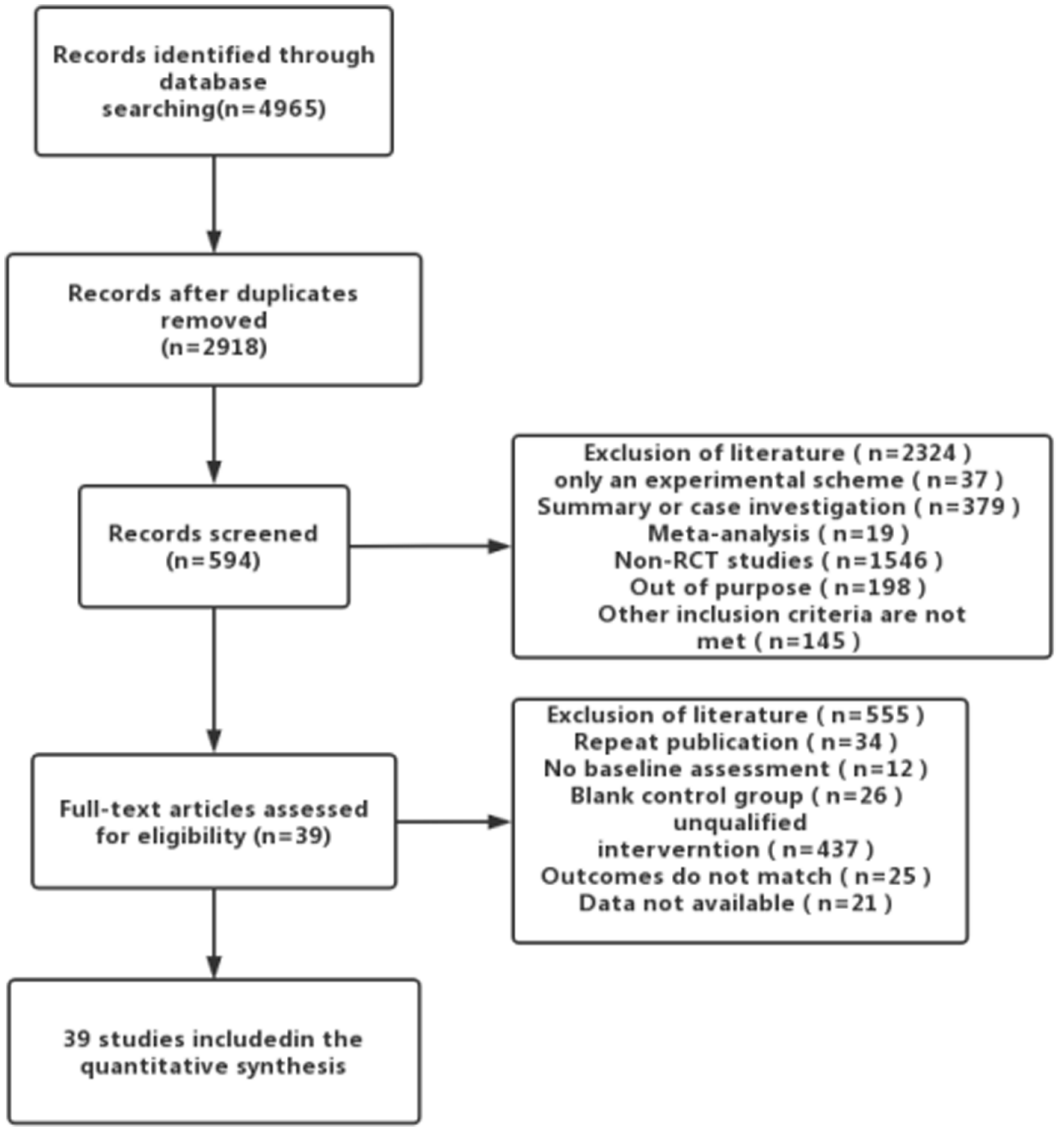
Process of literature search results and study selection.

Literature screening and data extraction were carried out independently by two professionally trained researchers, and the results were cross-checked by two persons. In case of disagreement or dispute, they were resolved through discussion or consultation with a third party. Data extraction included author, year, age, sample size, intervention measures, course of treatment, outcome indicators, adverse reactions, and literature type. The study selection process is illustrated in Figure 1.

#### 2.6.2. Data extraction and management

According to the bias risk assessment tool recommended by the Cochrane Handbook 5.1, our team members conducted a bias analysis of the included articles. As acupuncture and tuina are non-drug treatments, some participants and researchers involved in these studies could not be blinded (Table 2).

**Table 2:** Result of data extraction and management.

	Study ID	Random sequence generation selection bias	Allocation concealment selection bias	Blinding of participants and personnel performance bias	Blinding of outcome assessment detection bias	Incomplete outcome data attrition bias	Selective reporting bias	Other bias	Weight
1	Shuai Sun 2021	Low	Unclear	Unclear	Unclear	Low	Unclear	Low	60
2	Zhonghua Qin 2017	Low	Unclear	Unclear	Unclear	Low	Unclear	Low	110
3	Yiyi Zeng 2016	High	Unclear	Unclear	Unclear	Low	Unclear	Low	60
4	Jinng Huang 2022	Low	Unclear	Unclear	Unclear	Low	Unclear	Low	70
5	Xueyuan Jiang 2009	Unclear	Unclear	Unclear	Unclear	Low	Unclear	Low	70
6	Lili Zhang 2021	Low	Low	Unclear	Unclear	Low	Unclear	Low	60
7	Wang Luo 2021	Low	Low	Unclear	Unclear	Low	Unclear	Low	60
8	Tao Xu 2014	Low	Low	Unclear	Unclear	Low	Unclear	Low	140
9	Xiaocheng Shi 2012	Unclear	Unclear	Unclear	Unclear	Low	Unclear	Low	84
10	Xiaowei Wei 2005	High	Unclear	Unclear	Unclear	Low	Unclear	Low	120
11	Wei Wang 2019	Low	Unclear	Unclear	Unclear	Low	Unclear	Low	89
12	Ying Tang 2015	Low	Unclear	Unclear	Unclear	Low	Unclear	Low	47
13	Liping Cui 2021	Low	Unclear	Unclear	Unclear	Low	Unclear	Low	68
14	Lingzhi Wu 2018	Low	Low	Unclear	Unclear	Low	Unclear	Low	65
15	Wenqing Zou 2011	High	Low	Unclear	Unclear	Low	Unclear	Low	65
16	Yan Li 2021	Unclear	Low	Unclear	Unclear	Low	Unclear	Low	64
17	Xiaoshu Xie 2021	Low	Low	Unclear	Unclear	Low	Unclear	Low	100
18	Qiang Li 2019	Unclear	Unclear	Unclear	Unclear	Low	Unclear	Low	65
19	Qinghua Zhu 2022	Low	Unclear	Unclear	Unclear	Low	Unclear	Low	66
20	Lingzhe Li 2021	Low	Unclear	Unclear	Unclear	Low	Unclear	Low	80
21	Kaipeng Liang 2016	Unclear	Unclear	Unclear	Unclear	Low	Unclear	Low	149
22	Zusen Guo 2014	Unclear	Unclear	Unclear	Unclear	Low	Unclear	Low	60
23	Yu Xia 2022	Low	Unclear	Unclear	Unclear	Low	Unclear	Low	62
24	Lanzhi Huang 2019	Unclear	Unclear	Unclear	Unclear	Low	Unclear	Low	60
25	Liping Shen 2019	Unclear	Unclear	Unclear	Unclear	Low	Unclear	Low	80
26	Yulin Chen 2016	Low	Unclear	Unclear	Unclear	Low	Unclear	Low	86
27	Lezhong He 2012	Unclear	Unclear	Unclear	Unclear	Low	Unclear	Low	56
28	Ning Xu 2005	High	Unclear	Unclear	Unclear	Low	Unclear	Low	110
29	Aiqun Mo 2018	Unclear	Unclear	Unclear	Unclear	Low	Unclear	Low	64
30	Xijuan Zhang 2015	Unclear	Unclear	Unclear	Unclear	Low	Unclear	Low	76
31	Chaying Hu 2022	Unclear	Unclear	Unclear	Unclear	Low	Unclear	Low	48
32	Yan Zhang 2019	Low	Unclear	Unclear	Unclear	Low	Unclear	Low	70
33	Wei Ni 2017	Low	Unclear	Unclear	Unclear	Low	Unclear	Low	57
34	Dongwei Sun 2005	High	Unclear	Unclear	Unclear	Low	Unclear	Low	49
35	Haisheng Wu 2021	Low	Unclear	Unclear	Unclear	Low	Unclear	Low	98
36	Tianhong Hu 2018	Unclear	Unclear	Unclear	Unclear	Low	Unclear	Low	80
37	Xiong Qian 2017	Unclear	Unclear	Unclear	Unclear	Low	Unclear	Low	72
38	Jianxi Wang 2013	Unclear	Unclear	Unclear	Unclear	Low	Unclear	Low	60
39	Donglan Ye 2013	Unclear	Unclear	Unclear	Unclear	Low	Unclear	Low	82

#### 2.6.3. Assessment of risk of bias

Two independent reviewers assessed the risk of bias using the Cochrane Collaboration’s bias risk assessment tool. Specifically, evaluators assessed the risk of bias associated with random sequence generation (selection bias), assignment hiding (selection bias), subject and researcher blindness (implementation bias), outcome rater blindness (detection bias), integrity of reported outcome data (loss of follow up bias), and selective outcome reporting (report bias). Any disagreements about the risk of bias were resolved through discussions within the review group.

### 2.7. Assessment of trial quality and statistical analysis

The Grades of Recommendations Assessment, Development and Evaluation (GRADE) classification was used to grade the six outcome indicators. Stata 14.2 software was used to perform mesh meta-analysis (Zhang et al., 2014). The interrelationship between the interventions was represented by evidence network plots. Continuous variables were represented by average difference (MD) and 95% confidence intervals, while dichotomous variables were represented by odds ratio (OR) and 95% CI. A network evidence graph was drawn for outcome indicators. When closed rings were formed in the network diagram, the inconsistency test was needed to be carried out, and the inconsistency factor (IF) was calculated. IF value and *p*-value were used to determine any inconsistency. IF results of direct and indirect comparisons are consistent; the results of IF are close to 0% and the 95% confidence interval (CI) starts at 0, with a *p*-value of >0.05. The cumulative ranking curve (SUCRA) and the surface under the cumulative ranking curve surface under the cumulative ranking curve (SUCRA) were compared for different interventions, and the cumulative ranking curve (0 ~ 100) was used to rank the efficacy of various interventions. The larger the value is and the larger the area under the curve, indicating that the intervention is more likely to be the best intervention (Salanti et al., 2011; Chaimani et al., 2013). Meanwhile, the comparison-correction funnel plot was drawn to determine whether there was a publication bias or a small sample effect (Dias et al., 2013).

## 3. Results

### 3.1. Characteristics of included studies

Overall, relevant studies were retrieved from the database, and 39 RCT experiments were finally included in the analysis after one study was removed. All the studies are from China. It involves four intervention measures: acupuncture + tuina, acupuncture + traditional Chinese medical herbs, ordinary acupuncture, and commonly used Western medicine (Table 3).

**Table 3:** Characteristics of included RCTs.

Age	Study population		Intervention		Period of treatment		Outcome measures	Adverse events		References
	T	C	T	C	T	C		T	C	
7.7 ± 2.4	8.9 ± 1.5	30	30	Acupuncture+tuina	Western medicine	4 Weeks	①②③⑤⑥⑧	1	6	Shuai (2020)
8.04 ± 2.56	8.05 ± 2.58	54	56	Acupuncture+tuina	Western medicine	24 Weeks	①②③④⑦⑧	0	9	Zhonghua et al. (2017)
4.87 ± 0.78	4.8 ± 0.76	30	30	Acupuncture+tuina	Western medicine	4 Weeks	③⑤⑦⑧	\	\	Yiyi et al. (2016)
7.43 ± 2.35	7.62 ± 2.42	34	36	Acupuncture+tuina	Western medicine	4 Weeks	①②④⑦⑧	0	3	Jing (2022)
8.23 ± 2.31	8.25 ± 1.26	40	30	Acupuncture+tuina	Western medicine	8 Weeks	①②⑥⑦⑧	\	\	Xueyuan (2009)
9.31 ± 2.14	8.67 ± 2.53	30	30	Acupuncture+Chinese medicine	Western medicine	8 Weeks	①②④⑤⑥⑧	\	\	Lili et al. (2021)
7.56 ± 3.32	7.48 ± 2.65	30	30	Acupuncture+Chinese medicine	Western medicine	12 Weeks	①②⑤⑧	0	6	Wang (2021)
8.12 ± 1.45	8.48 ± 3.26	70	70	Acupuncture+Chinese medicine	Western medicine	8 Weeks	①②⑤⑦⑧	\	\	Tao and Jingdong (2014)
6.62 ± 2.14	5.35 ± 2.33	40	40	Acupuncture+Chinese medicine	Western medicine	8 Weeks	①②⑤⑧	\	\	Li Liang and Xiang (2012)
8.51 ± 2.33	8.13 ± 2.32	42	42	Acupuncture+Chinese medicine	Western medicine	6 Weeks	①②⑧	0	6	Xiaocheng et al. (2012)
7.95 ± 2.65	7.46 ± 3.21	60	60	Acupuncture+Chinese medicine	Western medicine	12 Weeks	④⑦⑧	\	\	Xiaowei et al. (2005)
8.08 ± 3.16	8.17 ± 2.97	45	44	Acupuncture+Chinese medicine	Western medicine	12 Weeks	①②③⑥⑦⑧	\	\	Wei and Fei (2019)
7.65 ± 2.31	7.18 ± 2.15	25	22	Acupuncture+Chinese medicine	Western medicine	12 Weeks	④⑥⑧	4	6	Ying et al. (2015)
10.34 ± 3.64	10.38 ± 3.52	34	34	Acupuncture+Chinese medicine	Western medicine	12 Weeks	①④⑤⑦⑧	2	6	Liping and Ningning (2021)
6.58 ± 2.72	5.97 ± 2.48	33	32	Acupuncture+Chinese medicine	Western medicine	12 Weeks	①②③⑦⑧	2	0	Wu Lingzhi et al. (2018)
7.62 ± 3.45	7.45 ± 2.13	33	32	Acupuncture+Chinese medicine	Western medicine	4 Weeks	④⑤⑧	0	10	Wenqing (2011)
8.57 ± 1.96	7.51 ± 1.18	32	32	Acupuncture+Chinese medicine	Western medicine	12 Weeks	①②③⑦⑧	0	12	Yan et al. (2021)
8.05 ± 1.60	8.06 ± 1.58	50	50	Acupuncture+Chinese medicine	Western medicine	12 Weeks	①②③⑦⑧	1	10	Xiaoshu et al. (2021)
9.08 ± 2.10	9.74 ± 2.23	37	38	Acupuncture+Chinese medicine	Western medicine	12 Weeks	④⑤⑧	2	15	Qiang (2019)
9.61 ± 2.67	9.59 ± 2.64	34	32	Acupuncture+Chinese medicine	Western medicine	8 Weeks	①②③⑦⑧	\	\	Qinghua et al. (2022)
8.30 ± 3.53	8.25 ± 3.65	40	40	Acupuncture+Chinese medicine	Western medicine	8 Weeks	①②③⑧	2	8	Li Lingzhe et al. (2021)
8.21 ± 1.23	8.62 ± 1.53	78	71	Acupuncture+Chinese medicine	Western medicine	12 Weeks	①②③⑧	1	3	Kaipeng (2016)
7.15 ± 2.35	7.48 ± 3.41	30	30	Acupuncture+Chinese medicine	Western medicine	8 Weeks	①②③⑧	4	6	Zusen et al. (2014)
7.69 ± 2.17	8.21 ± 2.34	31	31	Acupuncture	Western medicine	4 Weeks	①②③	1	7	Xia and Hongnan (2022)
8.83 ± 2.49	9.47 ± 2.52	30	30	Acupuncture	Western medicine	4 Weeks	①②③	0	6	Lanzhi (2019)
9.23 ± 2.79	8.90 ± 2.45	40	40	Acupuncture	Western medicine	8 Weeks	⑤⑥⑦⑧	4	8	Liping (2019)
8.4 ± 3.3	9.6 ± 4.2	43	43	Acupuncture	Western medicine	4 Weeks	④⑤⑧	0	3	Yulin (2016)
7.84 ± 3.43	7.49 ± 1.68	29	27	Acupuncture	Western medicine	8 Weeks	①②③⑧	\	\	Lezhong et al. (2012)
8.1 ± 2.6	7.9 ± 2.3	55	46	Acupuncture	Western medicine	8 Weeks	④⑦⑧	\	\	Ning and Haizhu (2005)
8.54 ± 3.23	8.15 ± 1.58	23	23	Acupuncture	Western medicine	8 Weeks	①③④⑧	\	\	Aiqun and Guiling (2018)
7.62 ± 2.65	7.42 ± 1.20	44	43	Acupuncture	Western medicine	12 Weeks	④⑥⑦⑧	\	\	Xijuan et al. (2015)
7.80 ± 2.34	8.11 ± 1.98	24	24	Acupuncture	Western medicine	16 Weeks	①②③⑧	1	3	Chaying and Hui (2022)
9.23 ± 1.84	8.97 ± 1.74	35	35	Acupuncture	Western medicine	8 Weeks	①②⑧	\	\	Yan (2019)
7.52 ± 2.85	7.624 ± 2.36	29	28	Acupuncture	Western medicine	4 Weeks	①②③⑥⑦⑧	\	\	Wei (2016)
8.26 ± 1.25	8.45 ± 2.32	25	24	Acupuncture	Western medicine	4 Weeks	③⑥⑦⑧	\	\	Dongwei (2005)
8.45 ± 2.33	8.17 ± 2.24	49	49	Acupuncture	Western medicine	8 Weeks	⑥⑦⑧	1	6	Haisheng et al. (2021)
7.14 ± 2.51	7.46 ± 2.33	40	40	Acupuncture	Western medicine	8 Weeks	①②③⑧	5	5	Tianhong (2018)
7.44 ± 2.62	7.15 ± 2.42	37	35	Acupuncture	Western medicine	12 Weeks	⑥⑦⑧	\	\	Xiong et al. (2017)
7.58 ± 1.49	7.62 ± 3.28	30	30	Acupuncture	Western medicine	8 Weeks	①②⑤⑧	1	6	Jianxi (2013)
8.1 ± 2.5	8.1 ± 2.5	82	82	Acupuncture	Western medicine	4 Weeks	⑤⑦⑧	0	9	Donglan (2013)

① YGTSS motor twitch; ② YGTSS vocal twitch; ③ total score of YGTSS; ④ TCM Syndrome Score Scale—nod and shrug; ⑤ TCM Syndrome Score Scale—laryngeal vocalization; ⑥ TCM syndrome score scale—upset; ⑦ TCM Syndrome Score Scale—total score; ⑧ clinical effectiveness.

### 3.2. Assessment of risk of bias

**Figure 2:**
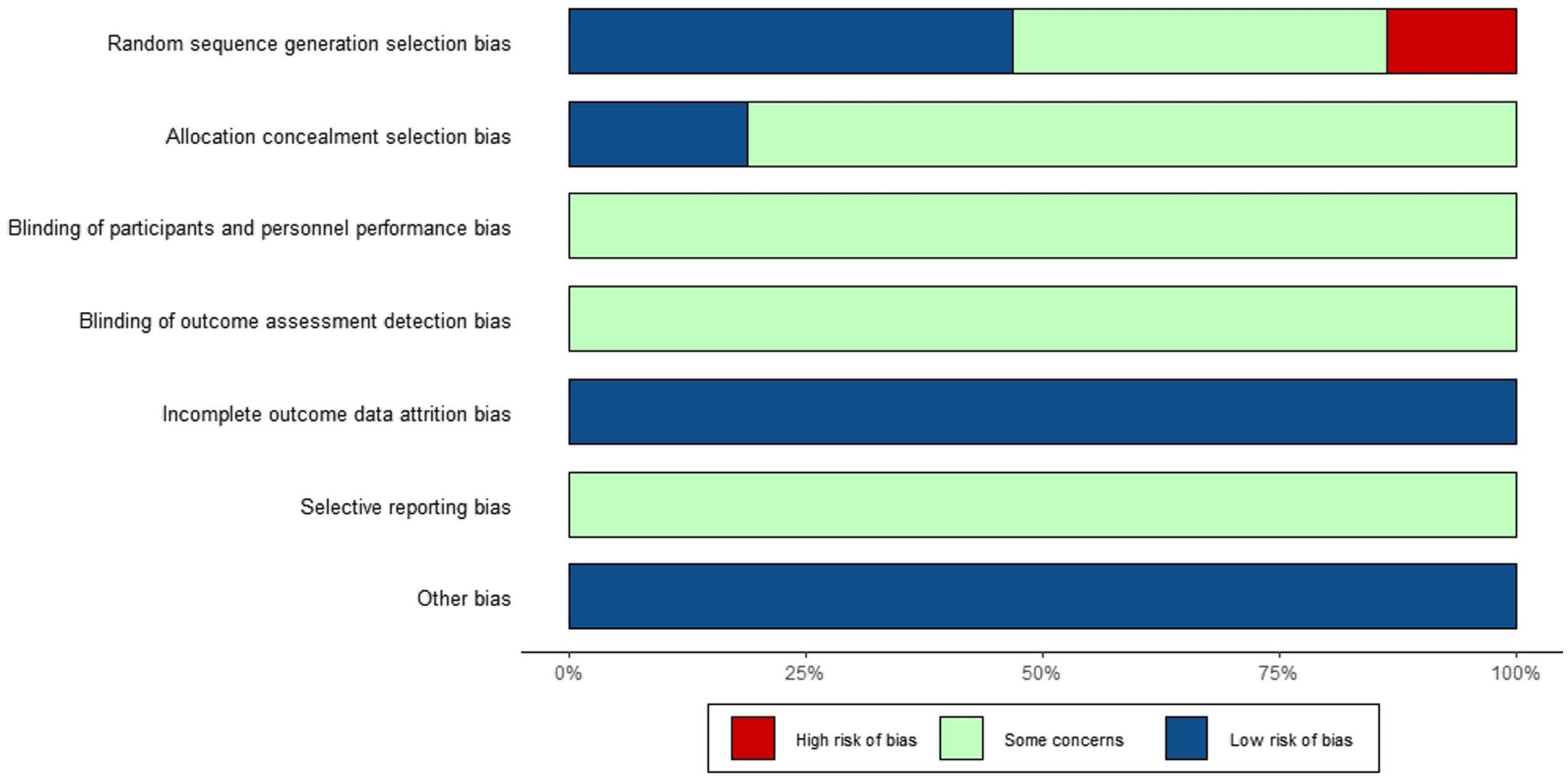
Risk of bias among included RCTs.

**Figure 3:**
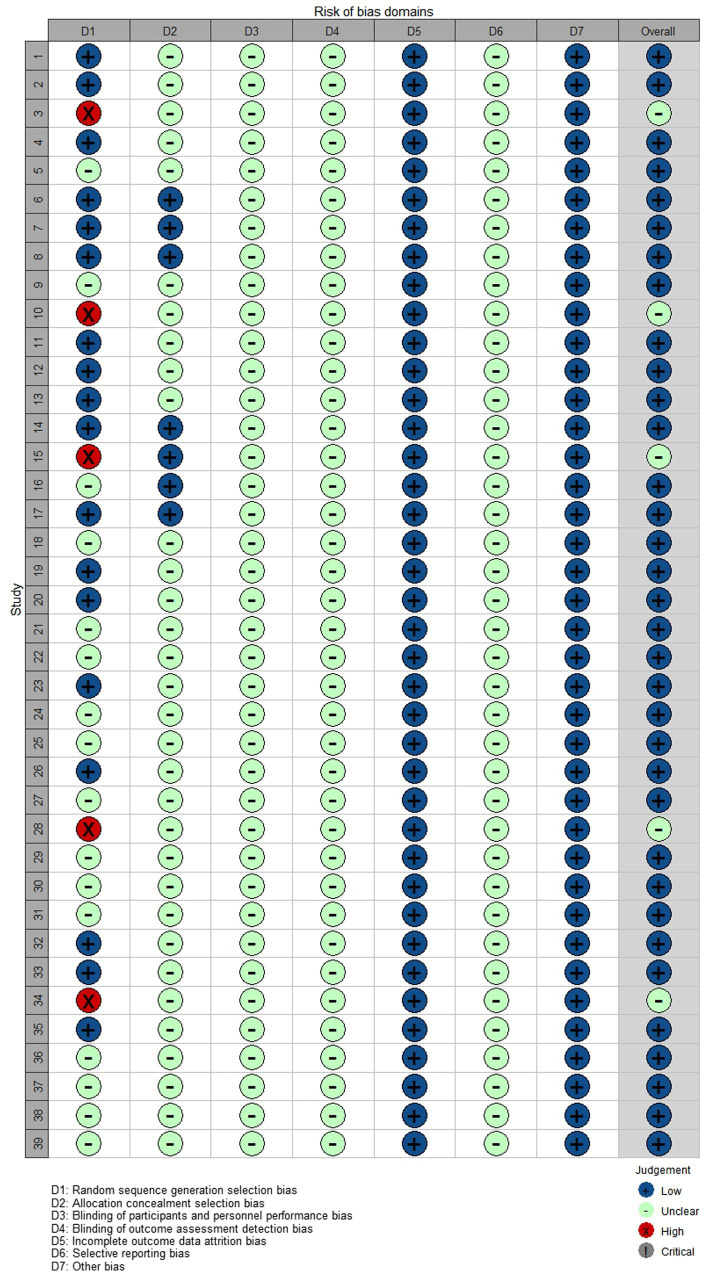
Risk of bias among included RCTs. Shuai Sun 2020 (1), Zhonghua Qin 2017 (2), Yiyi Zeng 2016 (3), Jinng Huang 2022 (4), Xueyuan Jiang 2009 (5), Lili Zhang 2021 (6), Wang Luo 2021 (7), Tao Xu 2014 (8), Xiaocheng Shi 2012 (9), Xiaowei Wei 2005 (10), Wei Wang 2019 (11), Ying Tang 2015 (12), Liping Cui 2021 (13), Lingzhi Wu 2018 (14), Wenqing Zou 2011 (15), Yan Li 2021 (16), Xiaoshu Xie 2021 (17), Qiang Li 2019 (18), Qinghua Zhu 2022 (19), Lingzhe Li 2021 (20), Kaipeng Liang 2016 (21), Zusen Guo 2014 (22), Yu Xia 2022 (23), Lanzhi Huang 2019 (24), Liping Shen 2019 (25), Yulin Chen 2016 (26), Lezhong He 2012 (27), Ning Xu 2005 (28), Aiqun Mo 2018 (29), Xijuan Zhang 2015 (30), Chaying Hu 2022 (31), Yan Zhang 2019 (32), Wei Ni 2017 (33), Dongwei Sun 2005 (34), Haisheng Wu 2021 (35), Tianhong Hu 2018 (36), Xiong Qian 2017 (37), Jianxi Wang 2013 (38), and Donglan Ye 2013 (39).

The risk analysis and summary of bias for all the studies included in this review are shown in Figures 2, 3.

### 3.3. Meta-analyses of the outcomes

#### 3.3.1. Results of pairwise meta-analyses

**Figure 4:**
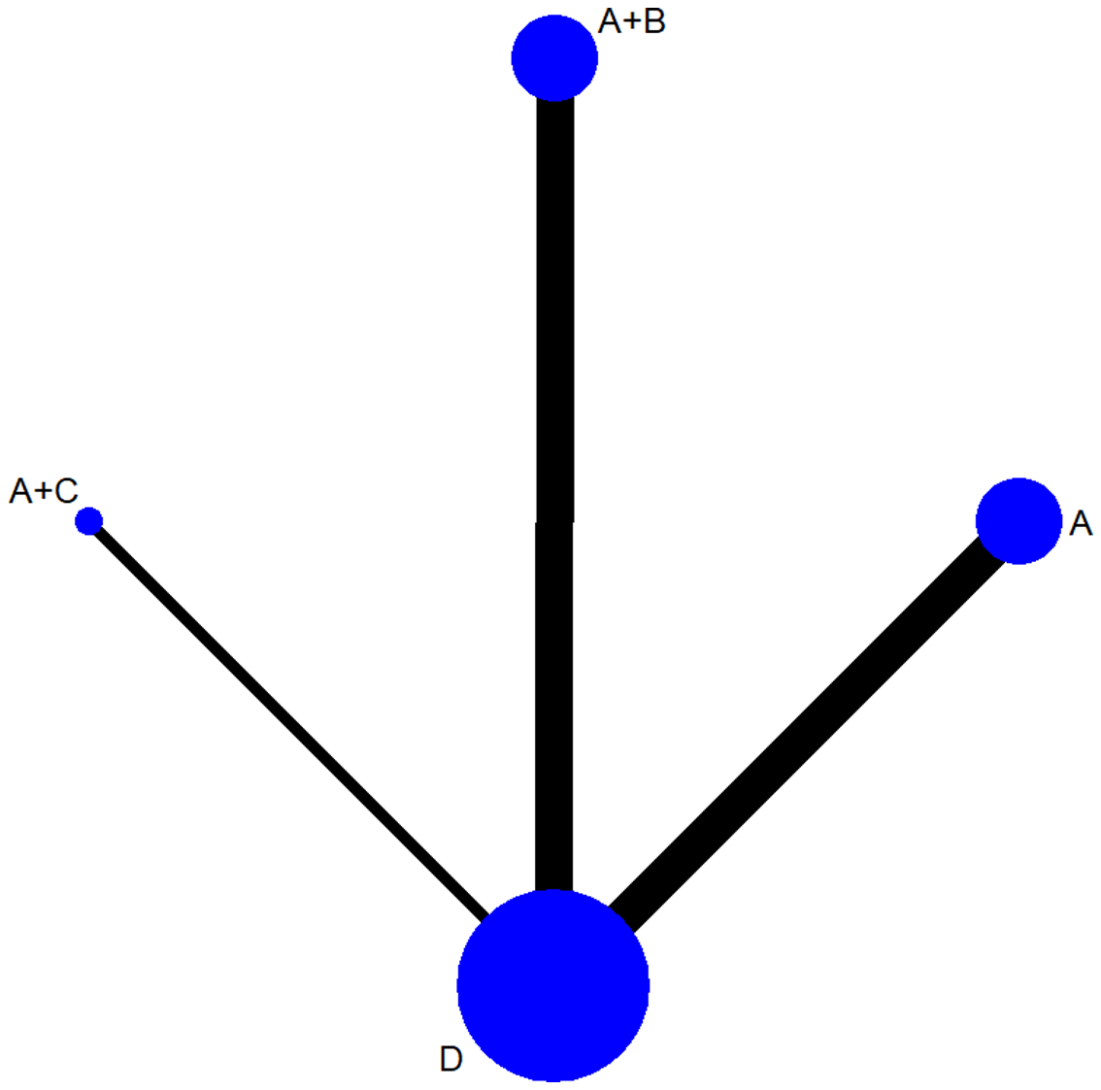
Results of pairwise meta-analyses. Acupuncture **(A)**, acupuncture + traditional Chinese medicine **(B)**, acupuncture + tuina **(C)**, western medicine **(D)**.

The results of Pairwise Meta-Analyses are shown in Figure 4. The interrelationship between the interventions was represented by evidence network plots. Continuous variables were represented by average difference (MD) and 95% confidence intervals, while dichotomous variables were represented by odds ratio (OR) and 95% CI. A network evidence graph was drawn for outcome indicators.

### 3.4. Primary outcome

#### 3.4.1. YGTSS-motor tics

A total of 24 articles reported changes in YGTSS motor twitch involving 1,767 patients. The mesh meta-analysis showed results of acupuncture + tuina (MD = 3.77, 95% CI [2.12, 5.42]), acupuncture + traditional Chinese medical herbs (MD = 4.90, 95% CI [3.69, 6.10]), and acupuncture (MD = 3.96, 95% CI [1.75, 6.17]) (Supplementary Table S1).

##### 3.4.1.1. Sorting of network meta-analysis results

The sorting of network meta-analysis results showed that acupuncture + traditional Chinese medical herbs (SUCRA = 87.3) > acupuncture + tuina (SUCRA = 59.6) > acupuncture (SUCRA = 53.1) (Supplementary Figure S1).

#### 3.4.2. YGTSS-vocal tics

A total of 21 pieces of literature reported changes in YGTSS vocal tic involving 1,626 patients. The reticular meta-analysis showed results of acupuncture + tuina (MD = 3.50, 95% CI [2.82, 4.18]), acupuncture + traditional Chinese medical herbs (MD = 4.03, 95% CI [3.50, 4.55]), and acupuncture (MD = 3.40, 95% CI [2.58, 4.22]) (Supplementary Table S2).

##### 3.4.2.1. Sorting of network meta-analysis results

The sorting of network meta-analysis results showed that acupuncture + traditional Chinese medical herbs (SUCRA = 92.6) > acupuncture (SUCRA = 56.5) > acupuncture = tuina (SUCRA = 50.9) (Supplementary Figure S2).

#### 3.4.3. YGTSS-total score

A total of 21 pieces of literature reported changes in the total score of YGTSS involving 1,649 patients. The mesh meta-analysis showed results of acupuncture + tuina (MD = 6.25, 95% CI [5.44, 7.05]), acupuncture + traditional Chinese medical herbs (MD = 6.98, 95% CI [6.31, 7.65]), and acupuncture (MD = 5.95, 95% CI [4.23, 7.68]) (Supplementary Table S3).

##### 3.4.3.1. Sorting of network meta-analysis results

The sorting of network meta-analysis results showed that acupuncture + traditional Chinese medical herbs (SUCRA = 92.3) > acupuncture (SUCRA = 57.4) > acupuncture + tuina (SUCRA = 50.3) (Supplementary Figure S3).

#### 3.4.4. Score for TCM syndrome-nod and shrug

A total of nine pieces of literature reported changes in score for the TCM syndrome score scale, nod and shrug, involving 712 patients. The reticular meta-analysis showed results of acupuncture + tuina (MD = 1.48, 95% CI [0.93, 2.03]), acupuncture + traditional Chinese medical herbs (MD = 1.73, 95% CI [1.16, 2.30]), and acupuncture (MD = 1.70, 95% CI [1.15, 2.26]) (Supplementary Table S4).

##### 3.4.4.1. Sorting of network meta-analysis results

The sorting of network meta-analysis results showed that acupuncture + traditional Chinese medical herbs (SUCRA = 75) > acupuncture + tuina (SUCRA = 73.1) > acupuncture (SUCRA = 51.9) (Supplementary Figure S4).

#### 3.4.5. Score for TCM syndrome-vocalization in the larynx

A total of 10 pieces of literature reported changes in the score for the TCM syndrome score scale, laryngeal vocalization, involving 804 patients. The reticular meta-analysis showed results of acupuncture + tuina (MD = 1.19, 95% CI [0.81, 1.57]), acupuncture + traditional Chinese medical herbs (MD = 1.34, 95% CI [0.88, 1.80]), acupuncture (MD = 1.50, 95% CI [1.06, 1.95]) (Supplementary Table S5).

##### 3.4.5.1. Sorting of network meta-analysis results

The sorting of network meta-analysis results showed that acupuncture + tuina (SUCRA = 84.7) > acupuncture + traditional Chinese medical herbs (SUCRA = 66.8) > acupuncture (SUCRA = 48.4) (Supplementary Figure S5).

#### 3.4.6. Score for TCM syndrome-vexation

A total of 10 pieces of literature reported changes in the score for the TCM syndrome score scale, upset, involving a total of 666 patients. The mesh meta-analysis showed results of acupuncture + tuina (MD = 0.98, 95% CI [0.44, 1.52]), acupuncture + traditional Chinese medical herbs (MD = 1.24, 95% CI [0.76, 1.72]), and acupuncture (MD = 1.17, 95% CI [0.62, 1.73]) (Supplementary Table S6).

##### 3.4.6.1. Sorting of network meta-analysis results

The sorting of network meta-analysis results showed that acupuncture + traditional Chinese medical herbs (SUCRA = 78.1) > acupuncture + tuina (SUCRA = 70) > acupuncture (SUCRA = 51.9) (Supplementary Figure S6).

#### 3.4.7. Score for TCM syndrome-primary symptom

A total of 15 pieces of literature reported changes in the score for the TCM syndrome score scale, main syndrome, involving a total of 1,174 patients. The results of the reticular meta-analysis showed that acupuncture + tuina (MD = 2.82, 95% CI [2.14, 3.51]), acupuncture + traditional Chinese medical herbs (MD = 4.09, 95% CI [3.45, 4.73]), and acupuncture (MD = 3.17, 95% CI [2.41, 3.93]) (Supplementary Table S7).

##### 3.4.7.1. Sorting of network meta-analysis results

The sorting of network meta-analysis results showed that acupuncture + traditional Chinese medical herbs (SUCRA = 98.6) > acupuncture + tuina(SUCRA = 59.4) > acupuncture (SUCRA = 42) (Supplementary Figure S7).

### 3.5. Secondary outcome

#### 3.5.1. Clinical efficiency

The clinical effective rate was reported in 39 pieces of literature, involving a total of 3,038 patients. The mesh meta-analysis showed results of acupuncture + tuina (OR = 1.97, 95% CI [1.10, 3.51]), acupuncture + traditional Chinese medical herbs (OR = 3.06, 95% CI [2.07, 4.51]), and acupuncture (OR = 1.72, 95% CI [1.29, 2.30]) (Supplementary Table S8).

##### 3.5.1.1. Sorting of network meta-analysis results

The sorting of network meta-analysis results showed that acupuncture + traditional Chinese medical herbs (SUCRA = 96.2) > acupuncture + tuina (SUCRA = 58) > acupuncture (SUCRA = 45.4) (Supplementary Figure S8).

### 3.6. Publication bias

**Figure 5:**
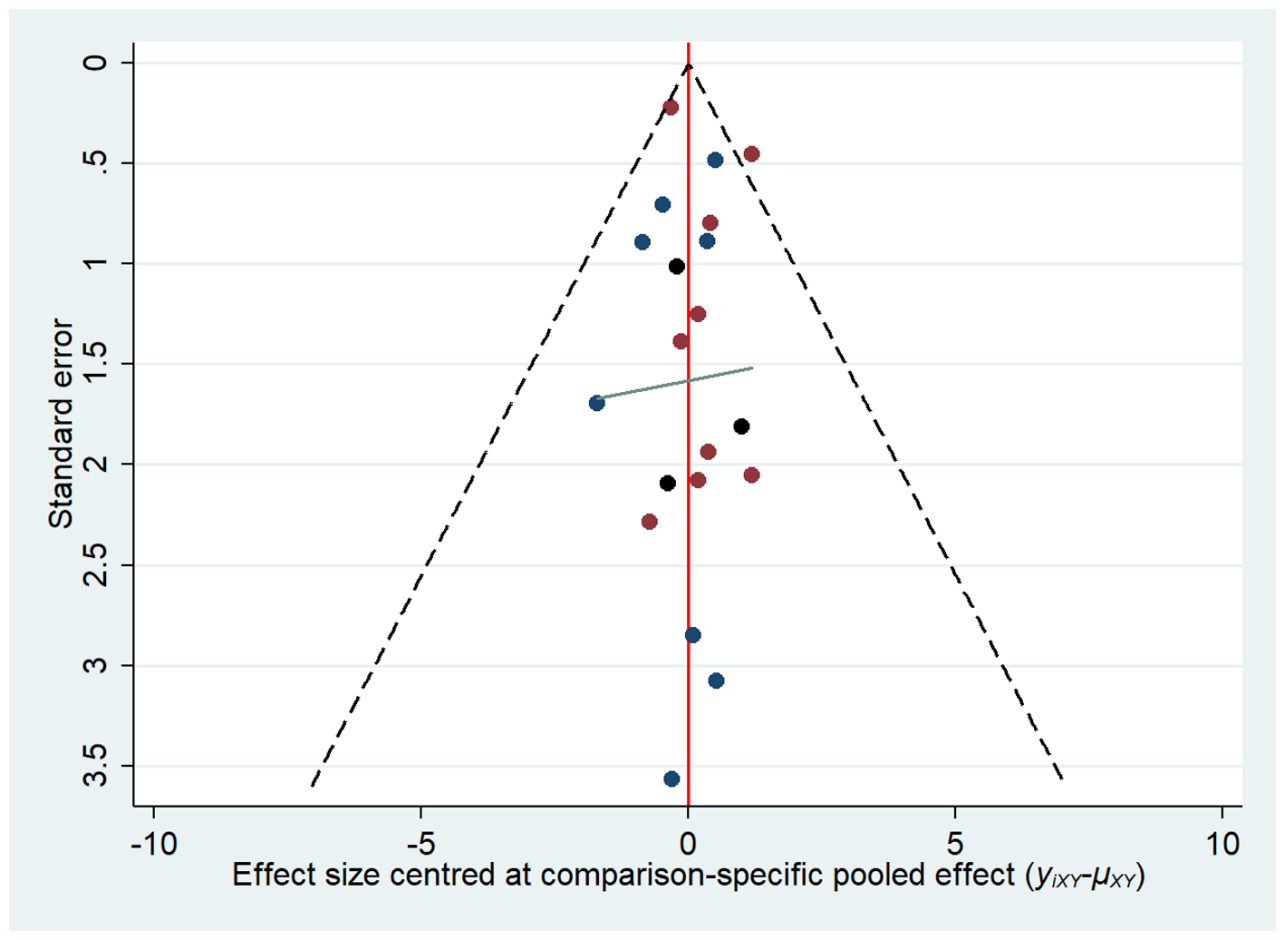
Results of publication bias.

In terms of the overall response rate, the heterogeneity detected in all included RCTs was low, so the fixed effects model was chosen for statistical analysis. Funnel plots were used to assess the potential role of publication bias in this review. All studies were symmetrically distributed around the X = 0 vertical line and the funnel plot was symmetric, indicating that there was no evidence of small-sample effects in the study network (Figure 5).

### 3.7. Adverse events

Of the 39 included studies, a total of 24 studies reported the occurrence of adverse events. A total of 50 patients had dizziness, 61 patients had nausea, 49 patients had drowsiness, 23 patients had fatigue, and eight patients had dry mouth. Among the five RCTs (acupuncture + tuina) and Western medicine, a total of four reported adverse reactions, with two cases in the experimental group and 23 cases in the control group. There were 16 cases of adverse reactions in the experimental group and 84 cases in the control group in the 17 RCTs of acupuncture + traditional Chinese medical herbs and Western medicine. In the 17 RCTs of acupuncture and Western medicine, there were 14 cases of adverse reactions reported in the experimental group and 52 cases in the control group.

### 3.8. GRADE classification of outcome indicators

The GRADE classification was used to grade the six outcome indicators. The results showed that the evidence of the YGTSS score, TCM syndrome score scale, and the clinical effective rate was rated as “medium” quality (Table 4).

**Table 4:** Results of GRADE classification of outcome indicators.

Comparison of acupuncture treatment for tic disorders in children			
Patient or population: pediatric tic disorder. Background: Any nationality, race or gender. Intervention: acupuncture + tuina, acupuncture + Chinese medicine, acupuncture. Control: Western medicine commonly used in clinic			
Index of outcome	Relative effect [MD (95%CI)]	Number of studies	GRADE
1. YGTSS Score Scale - Motor twitch	(Acupuncture+tuina) (MD = 3.77, 95%CI [2.12, 5.42]), (Acupuncture+Chinese medicine) (MD = 4.90, 95%CI [3.69, 6.10]), acupuncture (MD = 3.96, 95%CI [1.75, 6.17])	1,767 (24)	Middle +++⚪
2. YGTSS Score Scale - Vocal tics	(Acupuncture+tuina) (MD = 3.50, 95%CI [2.82, 4.18]), (acupuncture+Chinese medicine) (MD = 4.03, 95%CI [3.50, 4.55]), acupuncture (MD = 3.40, 95%CI [2.58, 4.22])	1,626 (21)	Middle +++⚪
3. YGTSS Score Scale - Total score	(Acupuncture+tuina) (MD = 6.25, 95%CI [5.44, 7.05]), (acupuncture+Chinese medicine) (MD = 6.98, 95%CI [6.31, 7.65]), acupuncture (MD = 5.95, 95%CI [4.23, 7.68])	1,649 (21)	Middle +++⚪
4. TCM Syndrome Score Scale - Nod and shrug	(Acupuncture+tuina) (MD = 1.48, 95%CI [0.93, 2.03]), (acupuncture+Chinese medicine) (MD = 1.73, 95%CI [1.16, 2.30]), acupuncture (MD = 1.70, 95%CI [1.15, 2.26])	712 (9)	Middle +++⚪
5. TCM Syndrome Score Scale - Vocalization in the larynx	(Acupuncture+tuina) (MD = 1.19, 95%CI [0.81, 1.57]), (acupuncture+Chinese medicine) (MD = 1.34, 95%CI [0.88, 1.80]), acupuncture (MD = 1.50, 95%CI [1.06, 1.95])	804 (10)	Middle +++⚪
6. TCM Syndrome Score Scale - Upset	(Acupuncture+tuina) (MD = 0.98, 95%CI [0.44, 1.52]), (acupuncture+Chinese medicine) (MD = 1.24, 95%CI [0.76, 1.72]), acupuncture (MD = 1.17, 95%CI [0.62, 1.73])	666 (10)	Middle +++⚪
7. TCM Syndrome Score Scale - Main disease	(Acupuncture+tuina) (MD = 2.82, 95%CI [2.14, 3.51]), (acupuncture+Chinese medicine) (MD = 4.09, 95%CI [3.45, 4.73]), acupuncture (MD = 3.17, 95%CI [2.41, 3.93])	1,174 (15)	Middle +++⚪
8. Clinical effectiveness	(Acupuncture+tuina) (OR = 1.97, 95%CI [1.10, 3.51]), (acupuncture+Chinese medicine) (OR = 3.06, 95%CI [2.07, 4.51]), acupuncture (OR = 1.72, 95%CI [1.29, 2.30])	3,038 (39)	Middle +++⚪

## 4. Discussion

### 4.1. Summary of the main results

A total of 39 RCT studies on acupuncture treatment of children with tic disorders were included in this study, and strict quality evaluation and risk assessment were carried out on the included studies. From the perspective of adverse reactions, compared to conventional Western drugs such as haloperidol, thiopiride, and risperidone, ordinary acupuncture or acupuncture combined with traditional Chinese medicine or acupuncture combined with massage have a lower incidence of adverse reactions, improving clinical treatment safety, and increasing clinical patient compliance. The scores of nodding and shrugging, laryngeal vocalization, upset, and TCM main symptoms in the TCM syndrome score scale were reduced. From the perspective of adverse reactions, compared with conventional Western drugs such as haloperidol, thioperide, and risperidone, both ordinary acupuncture itself, acupuncture combined with traditional Chinese medical herbs or acupuncture combined with tuina, have a lower incidence of adverse reactions, improving the clinical safety of the treatment and increasing the compliance of clinical patients.

The pathogenesis of tic disorder in children is complex, and multi-angle or multi-form combined treatment should be used in clinics. Acupuncture can effectively inhibit the occurrence of muscle twitching symptoms, and Chinese medicine can reduce the occurrence of diseases by regulating the abnormal state of the body (Guoxiang et al., 2017; Hanyuan and Zhenggang, 2017). The combination of acupuncture and traditional Chinese medical herbs has various forms, which is helpful to improve the clinical therapeutic effect, reduce the recurrence rate, and greatly improve the treatment compliance of patients and parents, thus improving the therapeutic effect.

The results of this study show that acupuncture combined with tuina, acupuncture combined with traditional Chinese medical herbs, and ordinary acupuncture can be used in the clinical treatment of children with tic disorder or as an adjunct therapy of conventional Western medicine and psychobehavioral therapy, which has a significant effect on improving the clinical efficacy of children with tic disorder and reducing the motor tic, vocal tic, and TCM syndrome score in YGTSS. Meanwhile, in terms of adverse reactions, acupuncture combined with tuina, acupuncture combined with traditional Chinese medical herbs, and ordinary acupuncture had better clinical safety than the conventional Western medicine group.

### 4.2. Comparison with other review studies

Previous similar meta-analyses (Ni Xinqiang et al., 2017; Runzhi et al., 2020; Lu et al., 2021; Jianrong et al., 2022) came to a conclusion similar to this systematic review that acupuncture or acupuncture combined with other therapies has better clinical efficacy than commonly used Western medicines in the treatment of tic disorders in children. However, this systematic evaluation has solved the unsolved problems of the previous systematic evaluation.

For example, previous systematic reviews only proved that acupuncture alone had better efficacy than commonly used Western medicine in the clinical treatment of tic disorders in children but did not make a horizontal comparison between acupuncture and acupuncture combined with other commonly used clinical traditional Chinese medical herbs therapies. In clinical treatment, acupuncture is not used as the only treatment means and is often supplemented by traditional Chinese medical herbs or tuina for combined treatment, so it will become very meaningful to explore the comparison of efficacy between them. In addition, GRADE was used in this system evaluation, and all the results were rated, which made this system evaluation more rigorous and the results more credible. At the same time, the time of inclusion of literature in this systematic evaluation is from the establishment date of each database to 15 November 2022, with a longer retrieval time limit.

### 4.3. Limitations of included studies

Although the aforementioned evidence can preliminarily indicate that acupuncture or acupuncture combined with tuina or traditional Chinese medical herbs can be used as clinically effective monotherapy or as an adjunct to conventional Western medicine and psychobehavioral therapy, we should still take a positive view of the shortcomings of the RCT articles included in this meta-analysis. (1) Most of the literature studies have random method errors or unclear descriptions, and most of the literature studies do not describe the blind method and distribution hiding, so there may be some bias in the results. (2) Due to different inclusion and exclusion criteria, the heterogeneity of some subgroup analyses in this study is large. At the same time, different RCTs may have different critical values of “recovery,” “obvious effect,” and “effective” for clinical effective rate, which will have a certain impact on the results of this study. (3) Fewer RCT references (acupuncture + tuina) were included than those acupuncture + traditional Chinese medical herbs and common acupuncture, which may not be sufficient to support the results of a meta-analysis. However, due to the low quality of some RCT experimental research methodologies included in this study, and the possibility of publication, selection, implementation, and other biases in some of them, a completely positive conclusion cannot be drawn. The efficacy and safety of acupuncture combined with tuina, acupuncture combined with traditional Chinese medical herbs, or ordinary acupuncture in the clinical treatment of children with tic disorders still need to be confirmed by more high-quality, multi-center, and large-sample size randomized controlled trials.

In addition, in the clinical treatment of tic disorders in children, it is also necessary to pay attention to the effect of the family intervention on the reduction of symptoms and the improvement of self-confidence in children with tic disorders (Nussey et al., 2013). Family tenacity is negatively correlated with family parental pressure (*p* < 0.01), suggesting that attention should be paid to the family environment of children while focusing on the treatment of the children themselves. The clinical nursing staff can conduct corresponding interviews with the main caregivers through communication, listening, and other ways to understand their physical and mental conditions and the difficulties in the treatment process and carry out the corresponding family nursing intervention in combination with the disease situation of the children to improve family resilience. At the same time, some studies have shown that (Wang et al., 2016) probiotics have certain effects on the function of the central nervous system and can improve behaviors related to mental diseases, such as anxiety, depression, autism spectrum disorder, obsessive–compulsive disorder, and memory ability. Therefore, acupuncture sometimes can also increase beneficial bacteria by soothing the spleen and stomach, regulating neurotransmitters by upregulating the abundance of bifidobacterium and lactobacillus, and playing a role in improving neuropsychiatric disorders.

## 5. Conclusion

The combination of acupuncture and traditional Chinese medical herbs can effectively improve the YGTSS score and TCM syndrome score scale of children with Tourette syndrome and has higher clinical efficacy compared with acupuncture and tuina or ordinary acupuncture. Therefore, the combination of acupuncture and traditional Chinese medical herbs may be the best therapeutic combination for the clinical treatment of children with tic disorders.

## Data availability statement

The original contributions presented in the study are included in the article/Supplementary material, further inquiries can be directed to the corresponding author.

## Author contributions

TP, HC, and WL conceived the study. TP and YL drafted the manuscript. TP, ZY, JZ, YL, and JY performed the literature search and collected the data. TP, ZY, and JZ analyzed and visualized the data. HC, JW, ZR, QS, XW, and WL helped with the final revision of this manuscript. All authors reviewed and approved the final manuscript.

## Funding

This study was funded by the Southwest Medical University and Affiliated Traditional Chinese Medicine Hospital of Southwest Medical University Joint Project (2020XYLH-021), the Key Project of Natural Science, and the Hunan Provincial Innovation and Entrepreneurship Training Program Project (002002028).

## Conflict of interest

The authors declare that the research was conducted in the absence of any commercial or financial relationships that could be construed as a potential conflict of interest.

## Publisher’s note

All claims expressed in this article are solely those of the authors and do not necessarily represent those of their affiliated organizations, or those of the publisher, the editors and the reviewers. Any product that may be evaluated in this article, or claim that may be made by its manufacturer, is not guaranteed or endorsed by the publisher.

## Footnotes

^1^ http://h-p.www.crd.york.ac.uk.hnucm.opac.vip/PROSPERO/
