# Supplementary figures and images for: Acupuncture and other traditional Chinese medicine therapies in the treatment of children’s tic syndrome: A network meta-analysis
Source: Front Neurosci. 2023 Apr 17;17:1156308. doi: 10.3389/fnins.2023.1156308 (PMC10153442; doi:10.3389/fnins.2023.1156308)

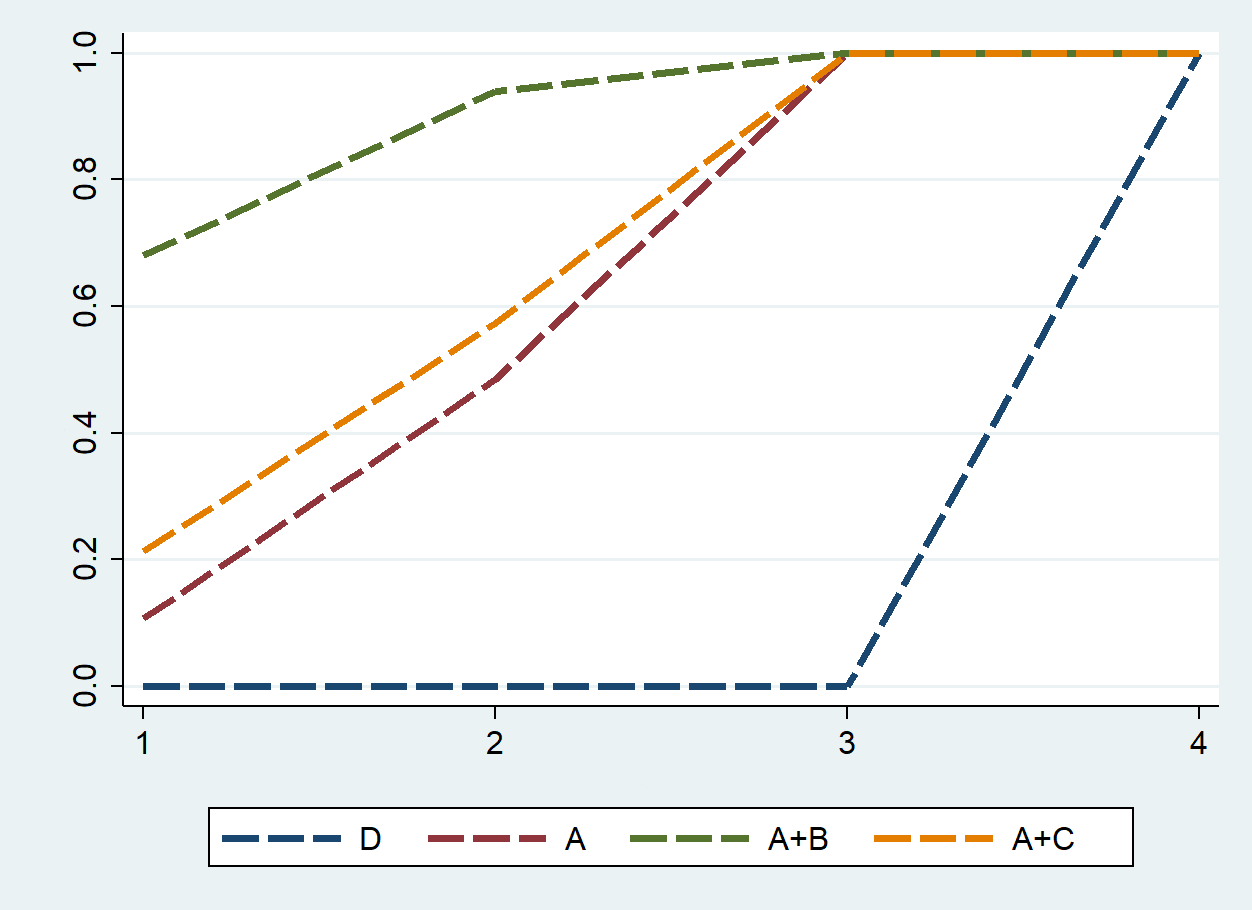

Supplement: Supplementary file 1 [file Image_1.TIF]

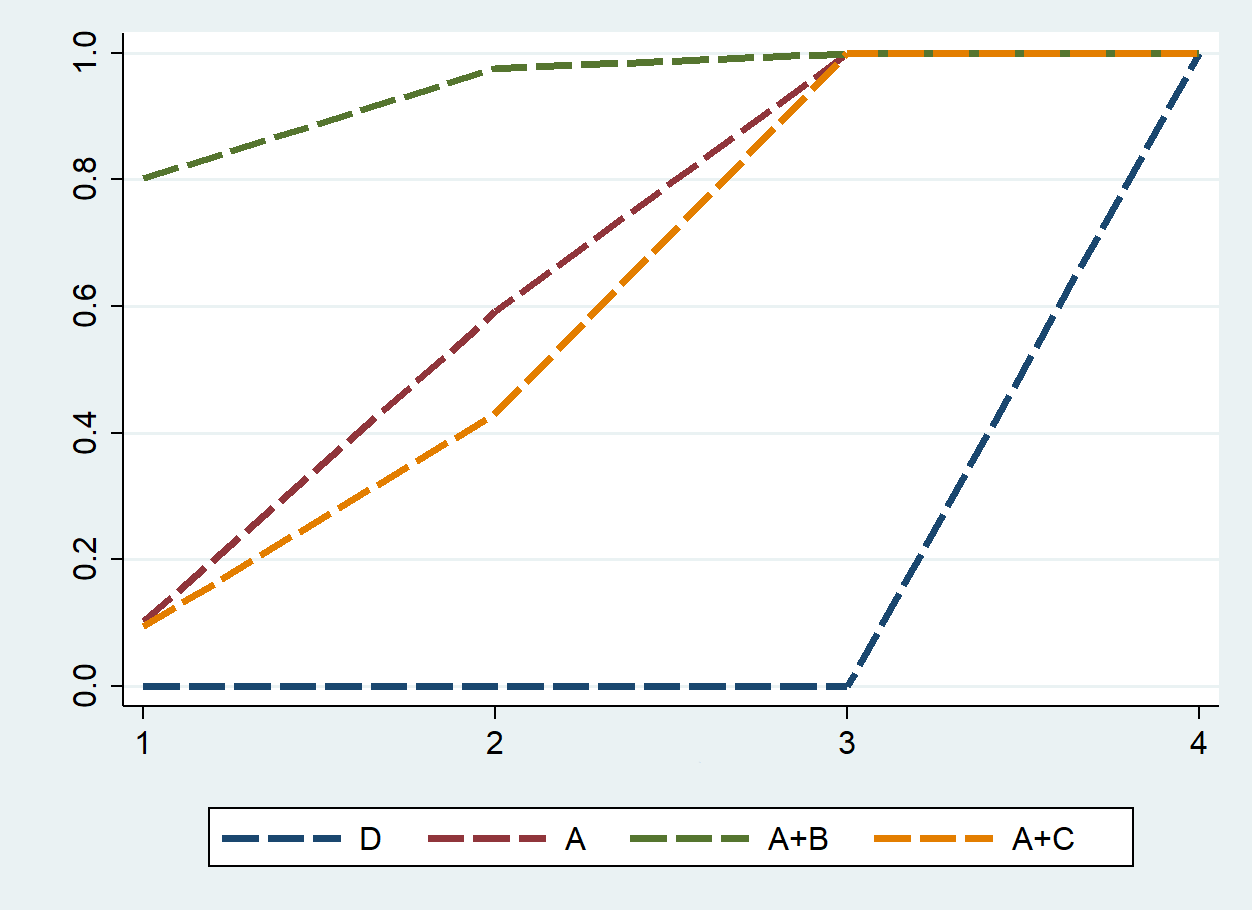

Supplement: Supplementary file 2 [file Image_2.TIF]

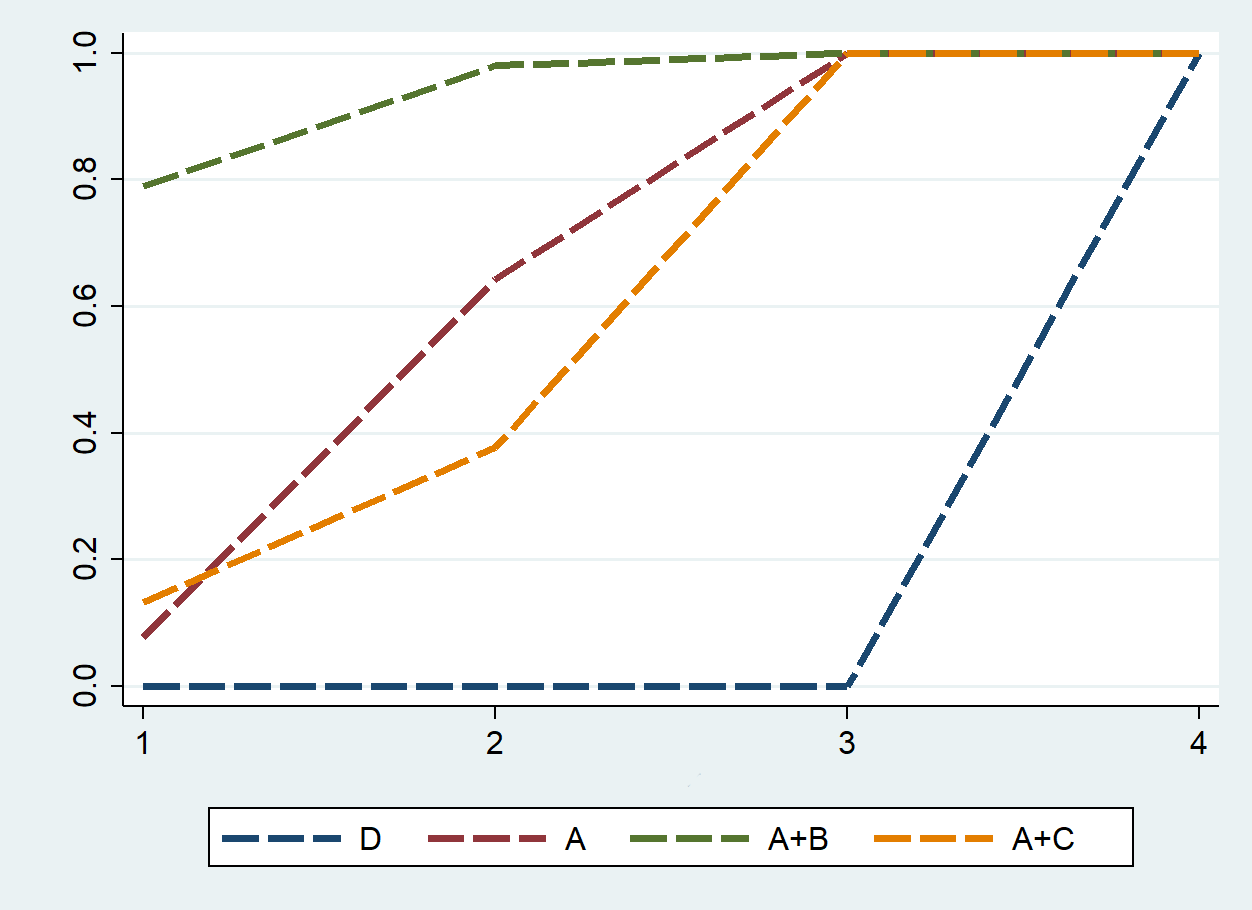

Supplement: Supplementary file 3 [file Image_3.TIF]

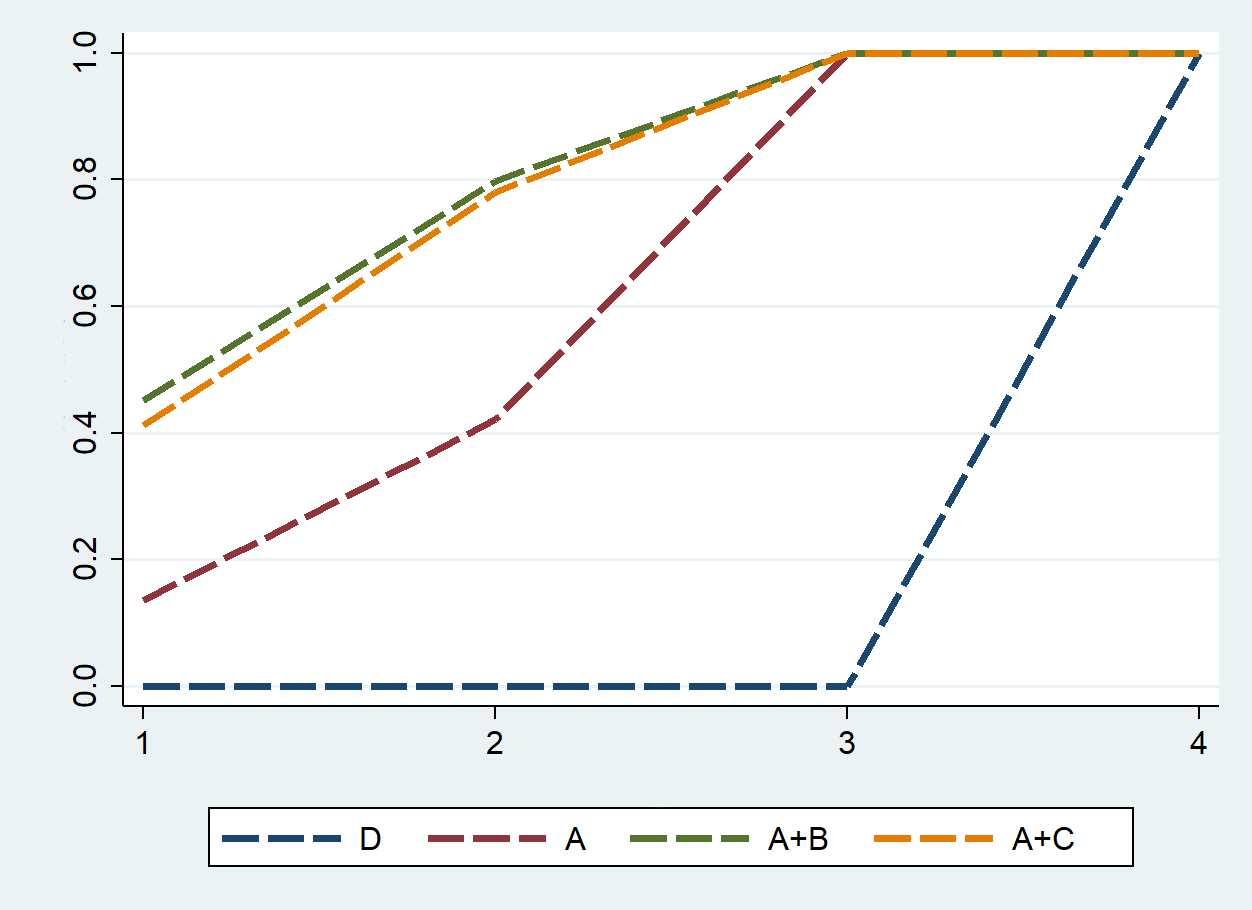

Supplement: Supplementary file 4 [file Image_4.TIF]

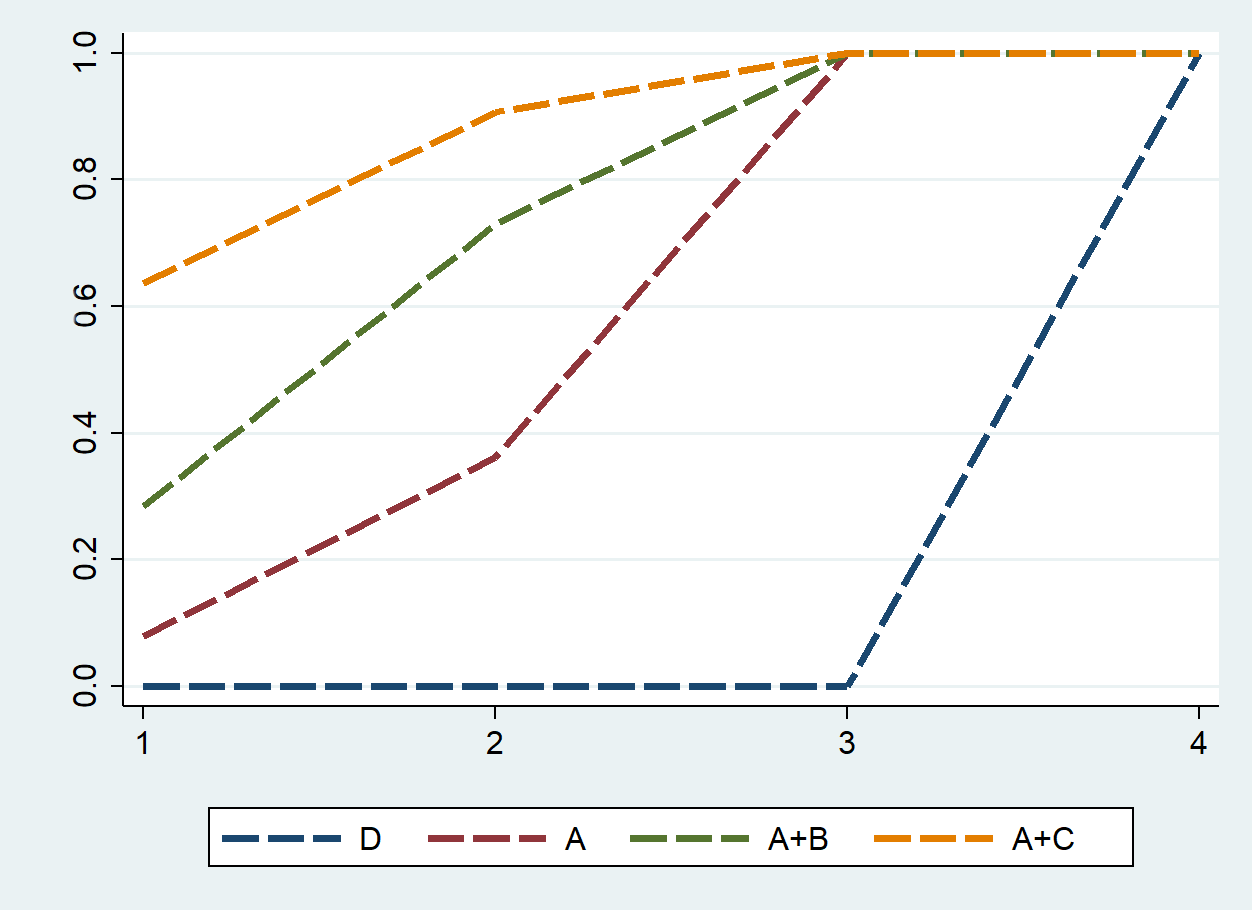

Supplement: Supplementary file 5 [file Image_5.TIF]

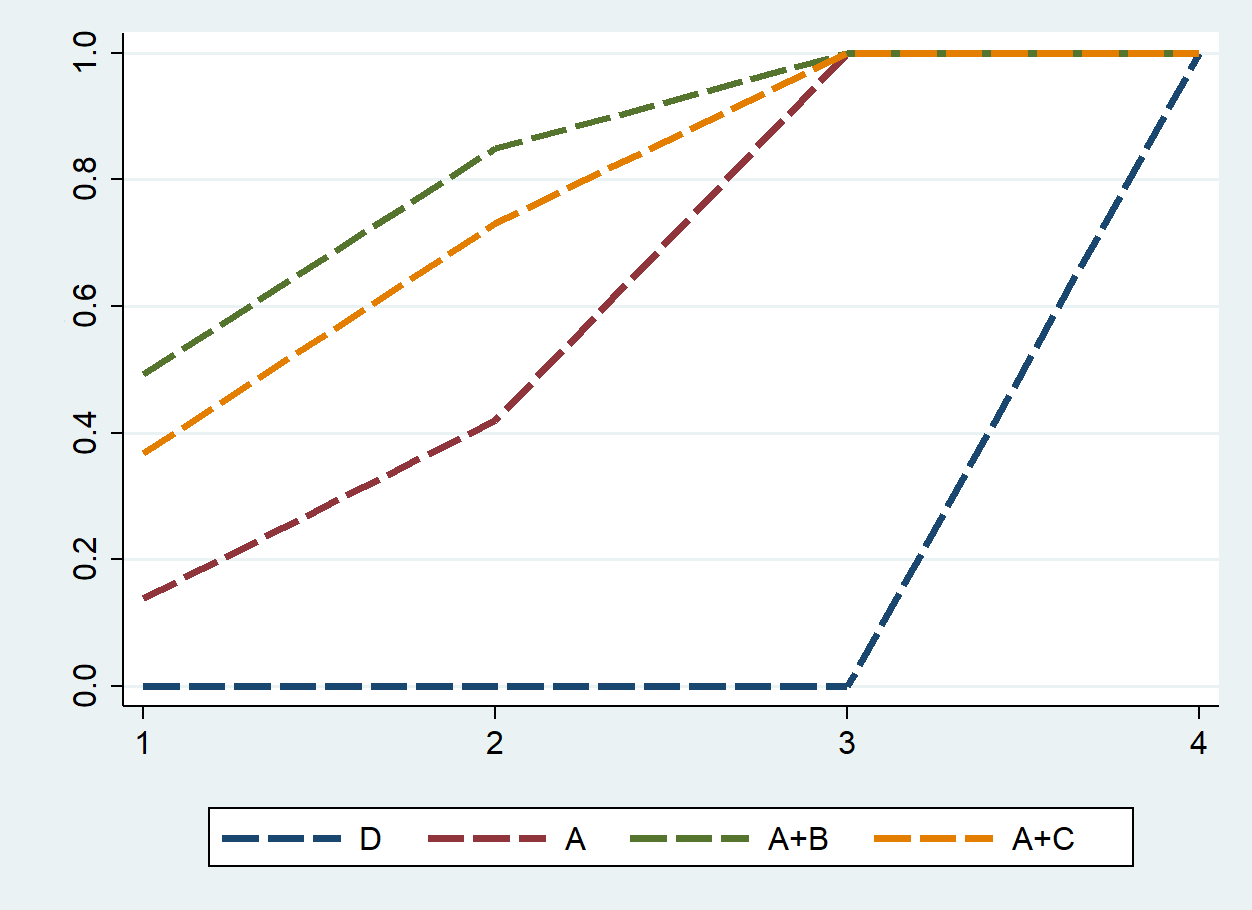

Supplement: Supplementary file 6 [file Image_6.TIF]

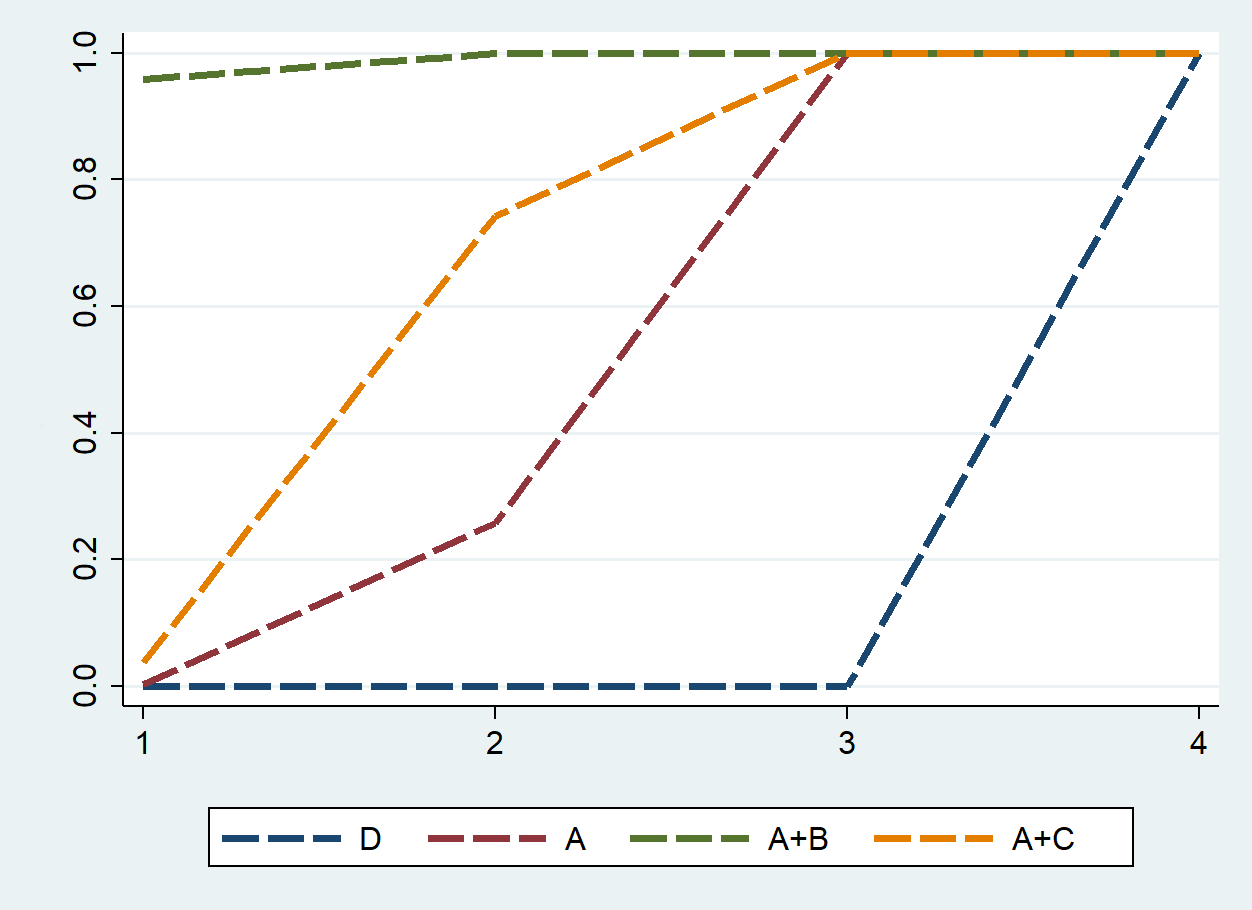

Supplement: Supplementary file 7 [file Image_7.TIF]

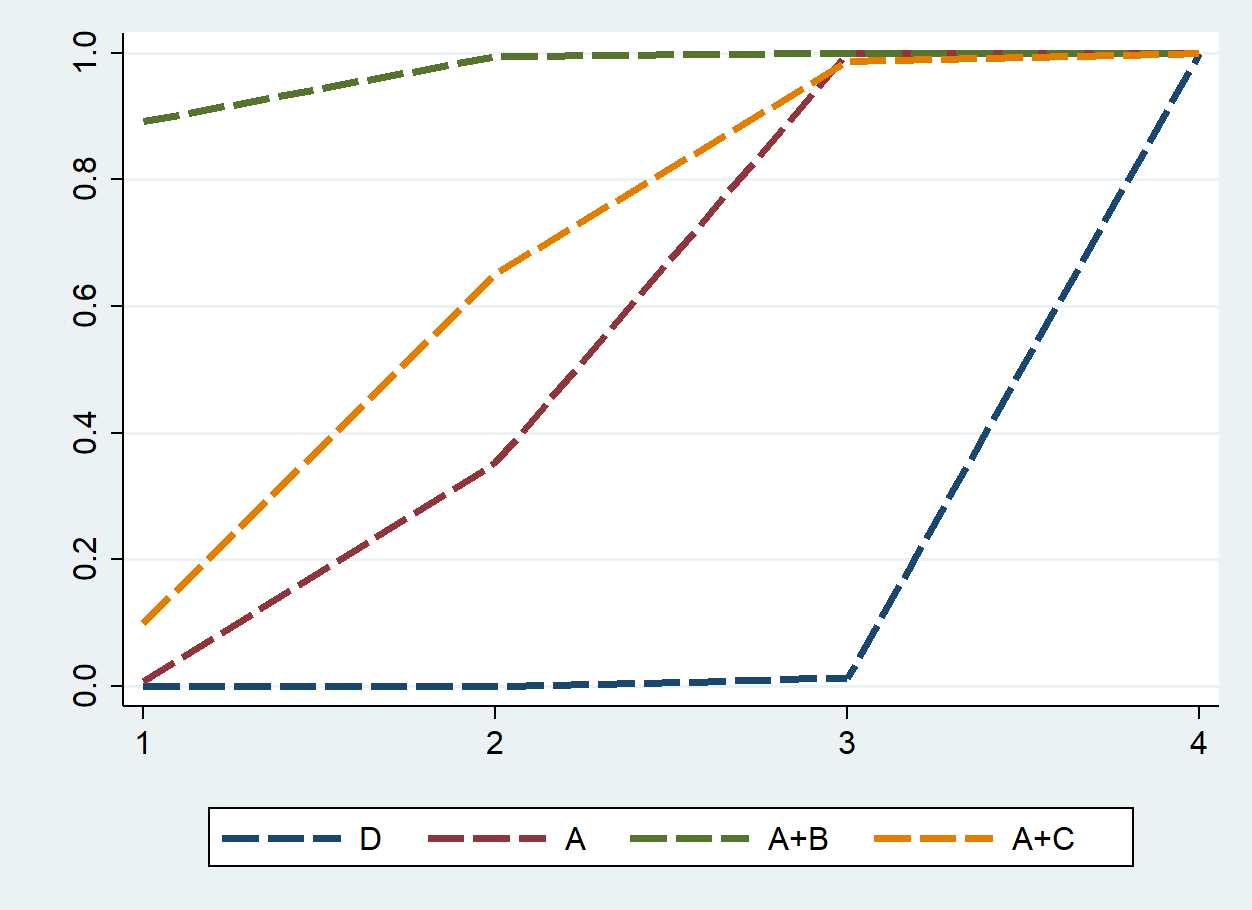

Supplement: Supplementary file 8 [file Image_8.TIF]
